# Acknowledgement to reviewers 2014

**DOI:** 10.1007/s11832-014-0628-6

**Published:** 2014-11-25

The Editors would like to thank the following reviewers for their continuous support during the year of 2014:

Franck Accadbled

Michael D. Aiona

Zaid Al-Aubaidi

Javier Albinana

Cristina Alves

Antonio Andreacchio

David D. Aronsson

Elhanan Bar-On

Jeremy Bauer

James Beaty

Michael J. Bell

Mohan V. Belthur

Michael K.D. Benson

Ernesto Bersusky

Victor Bialik

Rainer Georg Biedermann

John G. Birch

Robert Dale Blasier

Stephanie Boehm

Thomas Boeni

Silvio Boero

Gerard Bollini

Dieter Bolz

Christopher F. Bradish

Amichai Brezner

Reinald Brunner

Michael T. Busch

Carlo Camathias

Federico Canavese

Kelly Carmichael

Norris C. Carroll

Pablo Castañeda

Anthony Catterall

Henry Chambers

Tae-Joon Cho

Franck Chotel

Aurélien Courvoisier

Juan Carlos Couto

Alvin Howell Crawford

Robert Jay Cummings

Peter John Cundy

Jaroslaw Czubak

Charles d’Amato

Luciano Dias

John Dimitriou

Matthew B. Dobbs

John P. Dormans

Denis S. Drummond

Deborah Eastwood

Eric W. Edmonds

John Emans

Tomas Epeldegui

Thomas Erb

G. Ulrich Exner

Sharon Eylon

Eli Ezra

Guy Fabry

David S. Feldman

Frank Fitoussi

Edilson Forlin

Niklaus F. Friederich

Bruno Fuchs

Patricia Moraes Barros Fucs

Toshio Fujii

Julia F. Funk

Rudolf Ganger

Mark Gaston

Yael Gelfer

Ismat Ghanem

Werner Girsch

Joe Eric Gordon

Reinhard Graf

H. Kerr Graham

Alfred D. Grant

Franz Grill

Richard Henri Gross

Gunnar Hägglund

Carol Hasler

Dorian Hauke

Shlomo Hayek

Hanne Bastrup Hedin

Bernhard Heimkes

Anna Kathrin Hell

Yoram Hemo

John Anthony Herring

John E. Herzenberg

Sevan Hopyan

Gamal Hosny

James B. Hunter

Ivan Hvid

Talal Ibrahim

Ernesto Ippolito

Roger Jawish

Andrea Jester

Charles E. Johnston

Benjamin Joseph

Jean-Luc Jouve

Andre Kaelin

Lori Karol

Hidehiko Kawabata

Derek M. Kelly

David Keret

Sam Khamis

Mininder S. Kocher

Joseph Ivan Krajbich

Ruediger Krauspe

Andreas Krieg

Ken N. Kuo

Ron Lamdan

Erica Lamprecht

Mario Lampropulos

Lennart A. Landin

Pierre Lascombes

David Lebel

Seok Hyun Lee

Wallace Lehman

Joseph Lessing

Lynn Lindaman

David G. Little

Randall Loder

John E. Lonstein

Scott J. Luhmann

Nicolas Lutz

William G. Mackenzie

Eran Maman

Hans Michael Manner

Johannes Mayr

Terence McGuire

Charles T. Mehlman

A. Ludwig Meiss

Carlo Milani

Freeman Miller

Michael B. Millis

Kan Min

Bjarne Moeller-Madsen

Guy Molenaers

Jose Morcuende

Vincent Mosca

Scott J. Mubarak

Andreas Marc Mueller

Kishore Mulpuri

Lucas Murnaghan

Yasuharu Nakashima

Marek Napiontek

Yrjänä Nietosvaara

Iulian Nusem

Avi Ohry

Hakan Omeroglu

Norman Y. Otsuka

Dror Ovadia

Klaus Parsch

Laura Montserrat Pérez López

Peter Pizzutillo

Thomas Pressel

Tamir Pritsch

George T. Rab

Christof Radler

Leonhard E. Ramseier

James E. Robb

Andreas Roposch

Yishai Rosenblatt

Erich Rutz

Ralph Sakkers

Wudbhav N Sankar

Dietrich Schlenzka

Perry L. Schoenecker

Astrid Schulze

Eitan Nathan Segev

Dalia A. Sepúlveda

Sheldon R. Simon

David L. Skaggs

Theddy Slongo

Hae Ryong Song

Paul D. Sponseller

Carl L. Stanitski

Robert P. Stanton

Daniel Studer

Michael D. Sussman

Marek Synder

Terje Terjesen

Tim Theologis

Stephen J. Tredwell

Christian Tschauner

Cosimo Tudisco

Anja Van Campenhout

Johannes A. van der Sluijs

Michael G. Vitale

Andrew Wainwright

Eric Wall

Stewart Walsh

John H. Wedge

Dennis S. Weiner

Stuart L. Weinstein

Dennis R. Wenger

Bettina Westhoff

Roger F. Widmann

Kaye E. Wilkins

R. Baxter Willis

Thomas Wirth

James G. Wright

Haruhisa Yanagida

Moshe Yaniv

Muharrem Yazici

Reinhard D. Zeller

Kai Ziebarth

